# Correction: Developing an advanced gut on chip model enabling the study of epithelial cell/fibroblast interactions

**DOI:** 10.1039/d3lc90020g

**Published:** 2023-02-28

**Authors:** Marine Verhulsel, Anthony Simon, Moencopi Bernheim-Dennery, Venkata Ram Gannavarapu, Lauriane Gérémie, Davide Ferraro, Denis Krndija, Laurence Talini, Jean-Louis Viovy, Danijela Matic Vignjevic, Stéphanie Descroix

**Affiliations:** a Institut Curie, CNRS, UMR 168, IPGG, PSL Research University 6 rue Jean Calvin F-75005 Paris France stephanie.descroix@curie.fr +33 (0)1 56 24 64 68; b Institut Curie, CNRS, UMR 144, PSL Research University 12 rue Lhomond F-75005 Paris France danijela.vignjevic@curie.fr +33 1 56 24 63 66; c CNRS, UMR 7615, ESPCI Paris, UPMC, Sorbonne-Universités, PSL Research University F-75005 Paris France

## Abstract

Correction for ‘Developing an advanced gut on chip model enabling the study of epithelial cell/fibroblast interactions’ by Marine Verhulsel *et al.*, *Lab Chip*, 2021, **21**, 365–377, https://doi.org/10.1039/d0lc00672f.

The authors regret the omission of a funding acknowledgement in the original article. This acknowledgement is given below.

Venkata Ram Gannavarapu would like to acknowledge support from the Institut Curie 3-i PhD Program under the Marie Skłodowska-Curie Grant Agreement No. 666003.

The Royal Society of Chemistry apologises for these errors and any consequent inconvenience to authors and readers.

## Supplementary Material

